# Genomic Health Literacy Interventions in Pediatrics: Scoping Review

**DOI:** 10.2196/26684

**Published:** 2021-12-24

**Authors:** Aarushi Gupta, Joseph A Cafazzo, Maarten J IJzerman, Joost F Swart, Sebastiaan Vastert, Nico M Wulffraat, Susanne Benseler, Deborah Marshall, Rae Yeung, Marinka Twilt

**Affiliations:** 1 Institute of Health Policy, Management and Evaluation Dalla Lana School of Public Health University of Toronto Toronto, ON Canada; 2 Centre of Global eHealth Innovation Techna Institute University Health Network Toronto, ON Canada; 3 Institute of Biomedical Engineering University of Toronto Toronto, ON Canada; 4 Department of Health and Technology and Services Research Faculty of Behavioural, Management and Social Sciences Technical Medical Centre, University of Twente Twente Netherlands; 5 Melbourne School of Population and Global Health University of Melbourne Melbourne Australia; 6 Division of Pediatrics, Department of Pediatric Rheumatology and Immunology Wilhelmina Children's Hospital University Medical Center Utrecht Utrecht Netherlands; 7 Faculty of Medicine Utrecht University Utrecht Netherlands; 8 Division of Rheumatology, Department of Pediatrics Alberta Children's Hospital Calgary, AB Canada; 9 Alberta Children's Hospital Research Institute University of Calgary Calgary, AB Canada; 10 Department of Pediatrics Cumming School of Medicine University of Calgary Calgary, AB Canada; 11 Department of Health Sciences Cumming School of Medicine University of Calgary Calgary, AB Canada; 12 Department of Medicine Cumming School of Medicine University of Calgary Calgary, AB Canada; 13 Division of Rheumatology Department of Pediatrics The Hospital for Sick Children Toronto, AB Canada; 14 Immunology and Institute of Medical Science University of Toronto Toronto, AB Canada

**Keywords:** pediatrics, patient education, genetics, genomics, mHealth, digital health, internet, genetic knowledge, genomic health literacy, children, adolescents

## Abstract

**Background:**

The emergence of genetic and genomic sequencing approaches for pediatric patients has raised questions about the genomic health literacy levels, attitudes toward receiving genomic information, and use of this information to inform treatment decisions by pediatric patients and their parents. However, the methods to educate pediatric patients and their parents about genomic concepts through digital health interventions have not been well-established.

**Objective:**

The primary objective of this scoping review is to investigate the current levels of genomic health literacy and the attitudes toward receiving genomic information among pediatric patients and their parents. The secondary aim is to investigate patient education interventions that aim to measure and increase genomic health literacy among pediatric patients and their parents. The findings from this review will be used to inform future digital health interventions for patient education.

**Methods:**

A scoping review using PRISMA-ScR (Preferred Reporting Items for Systematic Reviews and Meta-Analyses extension for Scoping Reviews) guidelines and protocols was completed using the following databases: MEDLINE, Embase, CINAHL, and Scopus. Our search strategy included genomic information inclusive of all genetic and genomic terms, pediatrics, and patient education. Inclusion criteria included the following: the study included genetic, genomic, or a combination of genetic and genomic information; the study population was pediatric (children and adolescents <18 years) and parents of patients with pediatric illnesses or only parents of patients with pediatric illnesses; the study included an assessment of the knowledge, attitudes, and intervention regarding genomic information; the study was conducted in the last 12 years between 2008 and 2020; and the study was in the English language. Descriptive data regarding study design, methodology, disease population, and key findings were extracted. All the findings were collated, categorized, and reported thematically.

**Results:**

Of the 4618 studies, 14 studies (n=6, 43% qualitative, n=6, 43% mixed methods, and n=2, 14% quantitative) were included. Key findings were based on the following 6 themes: knowledge of genomic concepts, use of the internet and social media for genomic information, use of genomic information for decision-making, hopes and attitudes toward receiving genomic information, experiences with genetic counseling, and interventions to improve genomic knowledge.

**Conclusions:**

This review identified that older age is related to the capacity of understanding genomic concepts, increased genomic health literacy levels, and the perceived ability to participate in decision-making related to genomic information. In addition, internet-searching plays a major role in obtaining genomic information and filling gaps in communication with health care providers. However, little is known about the capacity of pediatric patients and their parents to understand genomic information and make informed decisions based on the genomic information obtained. More research is required to inform digital health interventions and to leverage the leading best practices to educate these genomic concepts.

## Introduction

### Background

Recent scientific breakthroughs and technological advancements in personalized and precision medicine are changing the way we diagnose and treat diseases, leading to more precise, predictable, and powerful health care that is customized for the individual patient [1]. However, individualized diagnostic and treatment pathway development is expensive and introduces new aspects of patient engagement into the more traditional medical practice. Personalized and precision medicine and genome sequencing have gone hand in hand and become more widely available and incorporated into clinical pediatric and adolescent care, either in the context of routine patient care or research [2,3]. However, genetic counselors have indicated that they lack the relevant knowledge, confidence, and practical techniques to educate adolescents about genomic concepts [4]; some health professionals have also expressed uncertainty about the cognitive abilities of adolescents to understand genomic concepts [5].

Genomic health literacy is defined as the basic knowledge of genetic and genomic concepts and the capacity to obtain, process, understand, and use genomic information for health-related decision-making [6]. Studies have shown that children and adolescents have the desire to learn more about the genetic factors related to their illness and to be more involved in the decision-making process of their treatment [7-11]. Moreover, pediatric patients undergoing genomic sequencing and their parents have expressed the desire to learn about actionable genomic research results [12-14]. As such, the increasing number of clinical genetic tests, research endeavors that use exome and genome sequencing, and increasing professional opportunities in genomics (eg, bioinformatics and genetic counseling) for adolescents entering the workforce point to a need to develop educational material on genomics for young people [15]. A systematic review by McGill et al [7] found that although children and adolescents in the general community may have a basic understanding of genetic concepts such as inheritance, they generally lack a deeper knowledge of concepts related to genetics and genetic testing. Although a high level of genomic health literacy is unlikely in children and adolescents, it may be valuable for young people who are affected or at risk of genetic conditions to have a general understanding of genomic concepts [7].

Navigating through the transitional stages of childhood and adolescence with a genetic condition could lead to difficulties with autonomy, identity development, and self-esteem [16]. Moreover, results from genetic testing of a child may have implications for parents and other family members [17]. The American College of Medical Genetics and Genomics guidelines recommend that children as young as 8 years should be actively involved in the decision and interpretation of the clinical exome or genome sequencing process to the extent that they are considered cognitively capable, which includes the assent of the child whenever reasonable and respecting their preferences [18].

Research has highlighted both positive and negative implications for the psychological outcomes for those who undergo genetic testing [19], and the ethical implications for returning genetic information to children have been widely debated, especially in the context of informed consent for genetic and genomic testing [20]. This attention resonates with the presumptions that parents know what is in their children’s best interest and that minors are unable to provide informed consent [20,21]. As children age, they gain decision-making capacity and an understanding of health conditions [20]. Therefore, including children and adolescents to various degrees as they age in health decisions related to genomic information is important yet challenging.

### Goal of the Study

This review was conducted as formative research for the Understanding Childhood Arthritis Network (UCAN) team to inform the design of a digital health intervention: a genomics patient education feature for patients living with juvenile idiopathic arthritis (JIA). JIA is the most common childhood chronic rheumatic disease and has a prevalence of 16-150 cases per 100,000 population [22] and can have a negative impact on the health-related aspects of quality of life [23]. Children and adolescents with JIA experience physical symptoms such as stiffness, fatigue, and sleep impairments; emotional symptoms such as stress, anxiety, and depression; and reduced social interactions [23]. As the etiology of JIA is unknown and is currently attributed to different genetic and environmental factors, a variety of pharmacological therapies are used to manage symptoms [24]. Biological disease-modifying antirheumatic therapies that target specific cytokines involved in the inflammatory cascade, such as tumor necrosis factor-α inhibitors, interleukin-1 inhibitors, and interleukin-6 inhibitors, have greatly changed the outcomes and morbidity associated with JIA but are associated with high costs [25,26] and safety concerns such as the risk of infection [25,26].

**Figure 1:**
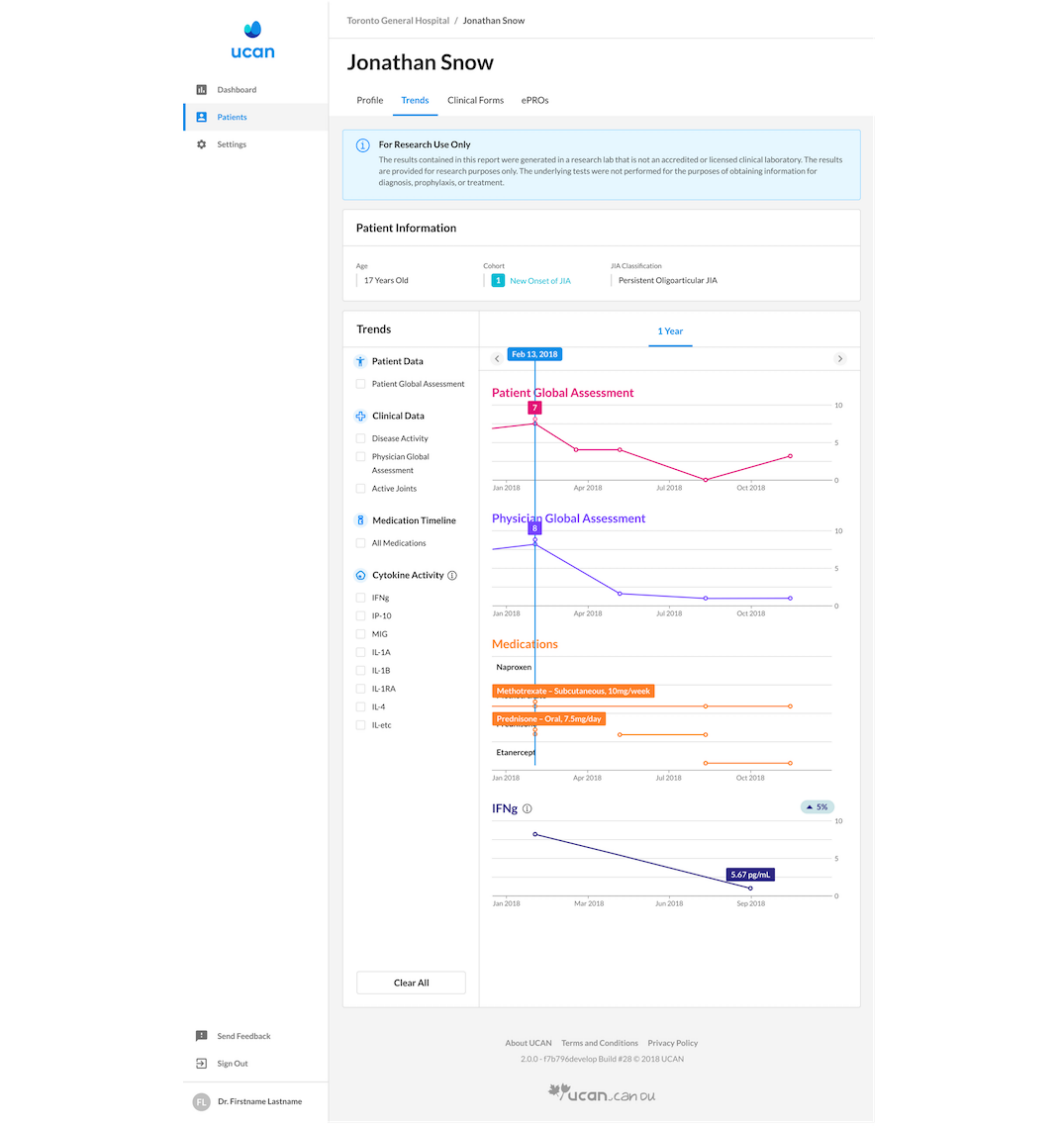
Understanding Childhood Arthritis Network platform genomics dashboard.

The ongoing UCAN study combines genomic discovery with patient-reported outcomes and health economic analyses to identify children at high risk of poor disease outcomes, define optimal ways to manage affected children, and develop a sustainable transdisciplinary network to improve the quality of life for all children with arthritis. One of the key features of the innovative UCAN platform is a novel genomics dashboard, which displays genomic information and trends in cytokine activity for patients. This tool acts as a visual aid for providers to discuss the severity of childhood arthritis with the patients with JIA and their parents and to identify the potential treatment targets based on the patient’s genomic profile (Figure 1).

In studies such as UCAN, where genomic information is shared with patients, there is a need to educate patients and their parents by improving the levels of genomic health literacy; to foster a better understanding of the disease; for meaningful conversation; for decision-making for disease management; and to foster a better understanding of implications on treatment, outcomes, quality of life, and long-term consequences. Although numerous studies have explored genomic health literacy among adult populations [27-29] and interventions to improve patient genetic education [30], there is a general lack of research regarding how children and adolescents understand genetic illnesses [7] and genetic and genomic testing, how such information should be conveyed to them, and what factors may affect communication efficacy [8]. In addition, no studies have investigated genomic health literacy or tools to educate the patients with JIA and their parents about genomic concepts.

### Objectives

We aim to perform a scoping review to identify and synthesize existing literature regarding the genomics health literacy levels and attitudes relative to receiving genomic information among pediatric patients and their parents to identify current practices and existing interventions that aim to improve genomic health literacy among pediatric patients and their parents. The term *genomics* is used as an umbrella term throughout the review as it encompasses the fields of genetic and genomic information; genomics describes the study of genes in their entirety, including their function, interaction, and environment and application of genome-based strategies [31]. Our research questions were intentionally broad to capture all the relevant literature relating to genomic health literacy and informational needs within pediatric patients:

- What are the genomic health literacy levels and attitudes toward receiving genomic information among pediatric patients and their parents?
- What interventions are known to educate pediatric patients and their parents about genomics to improve genomic health literacy levels and to facilitate a better understanding of their treatments?

## Methods

### Protocol and Registration

The protocol was not registered as scoping review protocols do not require registration. The scoping review methodology used was modeled based on the PRISMA-ScR (Preferred Reporting Items for Systematic Reviews and Meta-Analyses extension for Scoping Reviews) protocol [32]. This approach was used to study all the aspects of the topic, allow for a comprehensive exploration of patient knowledge and experiences, identify the existing literature relevant to the topics of interest, and identify gaps in the evidence.

### Eligibility Criteria

Studies were included if they met all of the following eligibility criteria: the study included genetic, genomic, or a combination of genetic and genomic information; the study population was pediatric (children and adolescents <18 years) and parents of pediatric patients or only parents of pediatric patients; the study included an assessment of the knowledge, attitudes, and understanding of genomic information; the study was conducted in the last 12 years between January 2008 and September 2020; the study only included human participants; and the study was in the English language. The time frame of the past 12 years was selected to capture the most recent and emerging practices in the genomics field.

### Information Sources

A formal electronic search and extraction was conducted between June 2020 and September 2020 on 4 electronic databases: MEDLINE, Embase, CINAHL, and Scopus. A gray literature search was also conducted on the following databases: Open Gray, CenterWatch, Cochrane Library, University of Toronto Libraries, TRIP database, ISRCTN registry, and advanced Google search.

Guidance and support from a faculty-affiliated librarian at the University of Toronto was received to formulate keywords and subject headings for the search strategy.

### Search Strategy

The search strategy included 3 main concepts: pediatrics, patient education, and genomics. Textbox 1 displays the full electronic search strategy used for MEDLINE. Multimedia Appendix 1 outlines corresponding searches for all the databases used during the search process for this scoping review.

**MEDLINE search strategy for the scoping review on genomic knowledge and education interventions in pediatrics inform digital health interventions.**

**Search strategy used for MEDLINE**

1. pediatric.mp. or exp Pediatrics/

2. paediatric*.tw,kf.

3. JIA.tw,kf.

4. ([pediatric or paediatric] adj5 illness).tw,kf.

5. “juvenile idiopathic arthritis”.tw,kf.

6. ([young or adolescen* or child*] adj5 illness).tw,kf.

8. autoimmune disease.mp. or exp Autoimmune Diseases/

9. Patient education.mp. or exp Patient education as Topic/

10. ([patient or young or adolescen* or child*] adj5 [educat* or learn* or knowledge or literacy or info*]).tw,kf.

11. “health knowledge”.tw,kf.

12. exp Genomics/

13. (genetic* or genomic* or genom* or biologic*).tw,kf.

14. exp Genetic Counseling/

15. (genetic adj5 [counselling or counseling]).tw,kf.

16. “genetic testing”.tw,kf.

17. “genom* sequencing”.tw,kf.

18. 1 or 2 or 3 or 4 or 5 or

19. 7 or 8 or 9

20. 10 or 11 or 12 or 13 or 14 or 15

21. 16 and 17 and 18

22. limit 19 to english

23. limit 20 to last 12 years

### Selection of Sources of Evidence

Duplicates were removed electronically, and the titles and abstracts were screened by 2 reviewers (AG and MT). Related articles that were removed during the screening process were stored in a reference list for relevant studies. The full-text screening was conducted (by AG and MT), and any discrepancies and disagreements were resolved by discussion and consensus. The most common reasons for exclusion of articles were that they involved a nonpediatric population (eg, health practitioners and adult patients), did not convey any genetic or genomic information to patients, did not have human participants, were not in the English language, were conference abstracts, were not published between 2008 and 2020, and were focused on disease etiology rather than genetic or genomic information.

### Data Items and Data Charting Process

To chart and extract data from the articles selected for the scoping review, an extraction criterion was developed by 2 reviewers (AG and MT) to extract information from each publication about the study country, city, urban or not urban geography, population, sample size, age of participants, pediatric disease types, duration, demographic information, design, methodology, journal of publication, and results of the study (Multimedia Appendix 2 [3,12,16,33-43]).

### Synthesis of Results

A thematic analysis of the nature and content of the articles was conducted to identify the common and recurring themes, topics, ideas, and patterns and to categorize the articles [44]. Both reviewers examined the data produced from charting and data extraction and identified the key codes relating to the research questions. The codes were used to summarize and report studies according to their main findings. A total of 6 major themes were identified during the thematic analysis: *knowledge of genomic concepts, use of the internet and social media for genomic information, use of genomic information for decision-making, hopes and attitudes toward receiving genomic information and support, experiences with genetic counseling, and interventions to improve knowledge of genomics.*

## Results

### Screening Process

A total of 4583 articles were identified from 4 electronic databases listed, and 35 articles were identified from a gray literature search. After the removal of 540 sets of duplicates, the remaining 3349 articles were screened according to the eligibility criteria. After the title and abstract screening stages, 41 articles were included in the full-text review and 27 articles were excluded owing to the reasons outlined in Multimedia Appendix 3. In total, 14 studies were included in the scoping review.

### Overview of the Included Studies

Of the 14 studies, 5 (36%) studies included populations where children were clinically diagnosed with various genetic conditions [16,33-36]; 4 (29%) studies had populations that were not diagnosed with an illness [12,37-39]; 1 (7%) study included children who were suspected to have a genetic condition but not diagnosed [40]; 1 (7%) study included children who were hospitalized for various reasons [3]; and the remaining 3 (21%) studies reported pediatric populations with illnesses such as cancer [41], congenital heart defects [42], and congenital lower limb deficiencies [43]. Most (11/14, 79%) of the studies selected for the review were from the United States [3,12,33-36,38-42], 14% (2/14) from Canada [16,43] and 7% (1/14) from the United Kingdom [37]. Table 1 presents the overview of the included studies.

**Table 1:** Overview of the studies included in the scoping review (N=14).

Theme and study			Population		Samples, n		Study design		Aims of the research
**Knowledge of genomic concepts**									
	Fitzgerald-Butt et al [42]	Parents of children with LVOT^a^		287		Quantitative		To examine the genetic knowledge and attitudes toward genetic testing of parents of children with heart defects affecting the LVOT	
	Gallo et al [34]	Parents in families in which the child has a single gene condition		142		Mixed methods		To identify unique patterns of information management and to explore the relationship between these patterns and individual and family characteristics and functioning	
	Lewis et al [37]	Children		539		Quantitative		To develop and validate a robust kids-KOGS^b^ suitable for use in the pediatric setting and for general public education	
	Rew et al [39]	Parents and adolescents		33 (22 adolescents and 11 parents)		Qualitative		To determine the levels of knowledge about genetics and approaches to decision- making related to genetic testing among adolescents and parents	
**Use of the internet and social media for genomic information**									
	Barton et al [33]	Parents of children (<18 years) who underwent genetic testing		20		Qualitative		To analyze parent views about the use of the internet and social media for informational and emotional support needs at different stages of their child’s genetic testing process	
	Roche et al [35]	Parents of children referred for genetic services		100		Qualitative		To investigate how parents of a child referred for genetic services search the internet for information before and after referral to a university pediatric genetics clinic, interpret and evaluate the information they obtained, and identify barriers that they encountered	
	Schaffer et al [36]	Mothers of children with genetic disorders		100		Qualitative		To investigate how mothers of children with genetic disorders use the internet to interpret, produce, and circulate genetic knowledge pertaining to their child’s condition; come to value their own experiential knowledge; and help shift the boundaries of what is considered as authoritative knowledge	
**Use of genomics information for decision-making**									
	McGowan et al [12]	Parents and adolescents (aged 13-18 years)		33 (15 adolescents and 18 parents)		Qualitative		To investigate decision preferences about values and involvement in choices of genomic sequencing results and to inform and guide practices of genomic researchers working with adolescents	
	Myers et al [38]	Parents and adolescents (aged 13-17 years)		326 (163 dyads)		Mixed methods		To examine decisions about learning genomic research results for the adolescents and whether choices were associated with demographic factors	
**Hopes and attitudes toward receiving information and support**									
	Campbell et al [43]	Parents of children with CLD^c^		25		Mixed methods		To collect data on Canadian pediatric patients affected by CLD followed to determine emotional supports, communication information, and implementation of genetics referrals	
	Khan et al [40]	Adults and parents of children with a suspected genetic condition		270, (191 adults and 79 parents)		Mixed methods		To investigate motivation and perceived resources to predict the amount and kinds of information that adult patients and parents of pediatric patients hoped to receive from diagnostic sequencing results	
**Experiences with genetic counseling**									
	Pichini et al [16]	Adolescents		11		Qualitative		To investigate the experiences and perspectives with respect to genetic counseling interactions and to understand adolescent-specific issues to better educate and support this population of patients	
**Interventions to improve knowledge of genomics**									
	Johnson et al [41]	Parents of children enrolled in the Genomes for Kids program; patients with cancer		121		Mixed methods		To determine whether a 2-step consent using a structured communication model would improve knowledge and understanding of key genetic concepts	
	Newcomb et al [3]	Children (aged 5-10 years) and parents		52 (26 children and 26 parents)		Mixed methods		To determine whether an original children’s book contributes to learning about the meaning of the terms *DNA* and *gene* in a sample of school-age children and whether experiencing the book with a pediatric nurse results in a better understanding of basic concepts than experiencing the book with a parent	

^a^LVOT: left ventricular outflow tract.

^b^A 10-item knowledge of genome sequencing measure for young people.

^c^CLD: congenital limb deficiency.

### Results of the Review

#### Knowledge of Genomic Concepts

Of the 14 studies, 4 (29%) studies investigated the knowledge and understanding of genetic concepts among pediatric patients or parents of pediatric patients [34,37,39,42]. These studies found varying results among participants of different age groups regarding the knowledge and understanding of genetic concepts including DNA, genome, genetic and environmental factors, the human genome project, and the sharing of genetic information.

Lewis et al [37] found that among school children between the ages of 11 and 15 years completing a 10-item kids-knowledge of genome sequencing measure for young people, the mean score was 4.24 (SD 2.49), on a scale where 0=low knowledge and 10=high knowledge. Age was also positively associated with the score in multivariate linear regression and the mean kids-knowledge of genome sequencing score was higher among girls than boys (4.44 vs 4.09, respectively; *t*_535_=1.61; *P*<.001). The most frequent correctly answered questions by children were related to DNA, such as *Our DNA is inside our cells* and *Our DNA doesn’t have an effect on how our body works* and the most frequent incorrectly answered questions were related to the genome, such as *Around 1% of our genome is the same as other people’s* and *Our complete set of DNA is called our genome*. 

Fitzgerald-Butt et al [42] tested genetic knowledge using a modified 18-item true or false instrument among parents of children with congenital heart defects and found that the mean genetic knowledge summary score was 73.8% correct. The most frequent correctly answered items were related to the interaction of genetic and environmental factors, such as *some diseases are caused by genes, environment, and lifestyle* (true; 97.2% correct) and *genes determine traits such as height, eye color and facial appearance* (true; 97.8% correct). The questions that the participants had the most difficulty with were related to basic genetic knowledge, such as identifying that *humans have 20 pairs of chromosomes* (false; 28% correct) and *parents pass both copies of each chromosome to their child* (false; 36.5% correct) are both false statements. Furthermore, educational attainment and household income were directly and significantly associated with genetic knowledge (*P*<.001).

Among parents and adolescents, Rew et al [39] found that although most participants had heard of genetic testing, the knowledge about the human genome project was generally lacking and inaccurate among younger adolescents (14-17 years), whereas older adolescents (18-21 years) demonstrated a better knowledge and accurate understanding of the human genome project. Most participants listed the internet and physicians as the sources of additional genetic information, and few participants listed books, articles, testing sites, teachers, and professional organizations as their sources.

Gallo et al [34] identified 4 unique information management patterns among parents of children (3-15 years) who have a single gene condition: *accurate understanding–open pattern* (30/86, 35%), *accurate understanding–selective pattern* (21/86, 24%), *discrepant understanding pattern* (13/86, 15%), and *confused understanding pattern* (22/86, 26%). In the accurate understanding*–*open and accurate understanding*–*selective (51/86, 59%) patterns, the parents had an accurate understanding of genetic concepts and were differentiated from one another based on their views about sharing information. The participants in the accurate understanding*–*open group actively sought information about conditions in addition to the information received from the health care providers (HCPs) and were open to sharing information about the child’s condition. On the other hand, participants in the accurate understanding*–*selective group struggled with sharing information about the child’s condition. In the discrepant understanding group, the parents within a family differed in the accuracy of their understanding of the genetic aspects of the condition and varied in their beliefs about seeking and sharing information. In the confused understanding group, the parents generally had an inaccurate understanding of one or more of the genetic aspects of the condition and some felt that they were unable to share information with others owing to a lack of understanding.

#### Use of Internet and Social Media for Genomic Information

Of the 14 studies, 3 (21%) studies investigated how parents of children with genetic disorders or children referred for genomic services searched the internet for information and emotional support about their child’s condition [33,35,36]. Barton et al [33] interviewed the parents of children who underwent genetic testing for clinical care, Roche et al [35] interviewed parents of children referred for genetic services, and Schaffer et al [36] interviewed mothers of children with genetic disorders.

Barton et al [33] reported that at each stage of the genetic testing process (ie, before testing, pending results, and after results), informational and support needs of the parents were different. Before testing, many parents had little knowledge of genetic testing or conditions; some parents said that knowledge of genetic conditions and testing was restricted to Down syndrome. The internet was used to explore the possible diagnoses or explanations for their child’s symptoms or challenges before testing, information about the genetic process during testing, and information about their child’s new diagnosis and possible treatments after testing [33].

All 3 studies found that parents search the internet to learn about the child’s condition, locate services for treatment, and find emotional support [33,35,36]. For example, Facebook groups [33], personal web pages, listservs, and chat rooms hosted by parent support groups [36] were mentioned as important resources to find support networks of families with similar experiences. Parents also found that searching using symptoms or diagnostic terms on widely available search engines such as Google or Yahoo or other websites sponsored by large health or advocacy groups (American Medical Association, Web MD, National Organization for Rare Disorders) played a key role in web-based searches [35]. Other targets for parents’ searches included preparing for the visit, learning about genetic testing options, diagnostic and prognostic information, management and treatment, finding clinical trials, and reading about research advances [33,35,36].

Roche et al [35] reported that the advantages of using the internet for information included convenience, feeling that clinicians were taking the parents more seriously, privacy, and the ability to find previously unobtainable information. In addition, Schaffer et al [36] reported that internet-searching allowed parents to gain traditional forms of scientific literacy, confidence in communicating with clinicians, and a sense of authority over genetic knowledge. Barriers to using the internet for information included emotional distress, unavailability of valid diagnosis to search for, discouragement from providers, misinformation, false hope, anxiety, concerns about the child’s privacy [33], keywords for searches, relevancy, and difficulty in remembering or spelling the diagnosis [35].

#### Use of Genomics Information for Decision-making

Of the 14 studies, 3 (21%) studies explored parents’ and adolescents’ preferences in decision-making in relation to genomic sequencing results [12,38,39]. These studies reported mixed feelings about receiving genomic results; some participants felt that it may be burdensome or raise privacy concerns [12], whereas others felt that it would help with future planning [12,38]. Myers et al [38] found that adolescents, in particular, expressed a desire to receive genomic information. However, adolescents were significantly less likely than parents to learn all results and that carrier status was the most frequent category that adolescents chose to learn about followed by adult-onset conditions, preventable conditions, and treatable conditions.

McGowan et al [12] found mixed perceptions among the participants regarding the adolescents’ capacity to participate in decision-making regarding genetic results [12]. In general, the participants agreed that participation in decision-making about the return of genomic research results should depend on age, maturity, and personality of the adolescent [12]. Regarding collaborative decision-making, parents felt that they should have the final say in decision-making about the return of genomic results. However, many adolescents felt that their decisional preferences would differ from their parents and that a collaborative decision-making model involving a health care representative could serve as an advocate for adolescents’ preferences.

Rew et al [39] found that when asked to make decisions about genetic testing, the mean age that young adolescents (aged 14-17 years) suggested was 16.6 years, whereas older adolescents (aged 18-21 years) suggested a higher mean age of 18.25 years. Almost half (45.5%) of the young adolescents also said that their parents would be their main source of information and advice related to decision-making regarding genetic testing.

#### Hopes and Attitudes Toward Receiving Genomic Information and Support

Of the 14 studies, 2 (14%) studies explored the hopes and attitudes toward receiving genomic information and support among parents of pediatric patients [40,43]. Khan et al [40] investigated the types of information used by adult patients and parents of pediatric patients who have a suspected genetic condition that has not been definitively explained or diagnosed. Campbell et al [43] explored the emotional supports, communication information, and implementation of genetic referrals among parents of children with congenital heart defects. Khan et al [40] found that the most common kinds of information that parents hoped to learn from diagnostic sequencing were the cause of illness (119/269, 44.2%), directions for illness management (98/269, 36.4%), diagnosis (72/269, 26.8%), disease risk for family members (62/269, 23%), helping others (31/269, 11.5%), advancing science (16/269, 5.9%), miscellaneous knowledge (14/269, 5.2%), prevention (8/269, 3%), and family planning (5/269, 1.9%).

Campbell et al [43] found that 16.7% of parents reported they were very satisfied, 33.3% were satisfied, 25% felt neutral, and 25% felt dissatisfied with the emotional support they received from their HCPs. Moreover, 80% of parents did not recall being referred to a support group by their HCP. When asked whether their child had been given a specific diagnosis, 48% of parents could not correctly recall their child’s specific diagnosis and 72% of parental classifications did not correspond to specific clinical classifications. In total, 56% of parents also reported that they sought additional information resources after talking to their HCP.

#### Experiences With Genetic Counseling

Of the 14 studies, 1 (7%) study investigated the experiences of genetic counseling for adolescents with a genetic condition [16]. The 3 main themes that emerged during interviews were understanding the genetic counselor’s role, increasing perceived personal control, and adolescent-specific factors influencing adaptation to one’s condition. The participants generally felt that they had a better understanding of what genetic counseling entailed and the distinct differences of a genetic counselor’s role from other HCPs after the session. This was owing to the discussion of biological pathways, inheritance and recurrence risk for future children, and preparation for the future during the session. In addition, all the participants felt that learning about genetics, inheritance, and origins of the condition was an important outcome of the genetic counseling session. In addition, genetic counseling helped the participants to contextualize the condition as part of their identity, receive anticipatory guidance about the future, and feel a higher sense of ownership and control over their health.

The main adolescent-specific factors that were reported to influence adaptation to one’s genetic condition were isolation, social connectedness, independence and privacy, and the timing of genetic counseling [16]. Adolescents noted feeling isolated as a result of their condition and being treated differently to others. In addition, the adolescents’ perspectives about their condition were influenced by social connectedness with family, peer groups, and the larger community of other individuals with the same condition. As such, the participants expressed a desire to fit in and be perceived as *normal* by their friends. Although adolescents sought social connectedness, they expressed a need for independence and privacy, particularly within the family construct. Finally, the participants stated that they felt a greater sense of stability during the middle school to high school period than during the elementary school period and felt that it was a more appropriate timing for genetic counseling.

When asked about the tools and strategies for genetic counseling practice, the participants suggested using video clips and animations on a computer or tablet to describe the inheritance or biological processes, the normalization of their condition, and the ability to choose whether parents are present for all or a part of the session. In addition, the adolescents suggested that the genetic counselor could assist them with identifying reliable resources to gather information about their condition, support groups, and a postsession letter highlighting the key points relevant to them.

#### Interventions to Improve the Knowledge of Genomics

Of the 14 studies, 2 (14%) studies investigated the impact of interventions in improving the knowledge and understanding of genetic concepts [3,41]. The results of these 2 studies are presented in Table 2. Both studies used the Genetic Knowledge Index (GKI) developed to assess lay knowledge of genetic concepts among a general population not known to be at risk for genetic disease and not exposed to genetic counseling or research involving genomics, to test the understanding of genetic concepts among participants [45].

**Table 2:** Overview of studies that investigated the impact of interventions on improving the knowledge and understanding of genetic concepts.

Study	Aims of the research	Pretest results	Posttest results
Newcomb et al [3]	Whether an original children’s book called “What DNA Does,” designed as a visual aid to assist in the assent process for children enrolling in genetic testing research, could increase the child’s and parent’s understanding about “DNA” and “genes” and whether children reading the book with a pediatric nurse would result in a better understanding of genetic concepts than reading the book with a parent.	Median GKI^a^ score was 4 (0=all incorrect and 5=all correct), with 54% of respondents making only 1 incorrect response; most difficult item: “Racial differences in academic ability are caused by genetics.” Both parents’ and child’s understanding of the terms was minimal before reading the book; no participants mentioned learning about genetics or DNA in school. A total of 65% (17/26) of parent respondents said they did not know what DNA was or stated vague or inaccurate definitions. None of the child respondents was able to explain DNA in simple terms, although some were able to repeat phrases they had heard. The primary recurring theme in the conversations about DNA before reading the book was “blood”; both children and parents expressed the idea that DNA is somehow closely related to or is a part of blood and that blood has something to do with human identity.	GKI was not completed after the test. After reading the book, most children had more articulate and accurate understandings of “DNA,” but no better understanding of its function; 2 children were more confused after reading than before. Children who read the book with a nurse had a better understanding of DNA’s function than those who read it with a parent. Increased accuracy of describing the meanings of DNA and gene was demonstrated by all the participants in the nurse–child–parent reading group and in two-third of the children in the parent–child reading group.
Johnson et al [41]	Whether a 2-step consent using a structured communication model would improve the knowledge and understanding of key genetic concepts among parents of children with cancer. The model involved a single study nurse who approached and obtained consents from the families with a standardized script, an informational cover sheet, and baseline pretest responses to educate parents on genetic concepts during the study introductory visit. At the subsequent informed consent visit, the nurse used a checklist and an informed consent document to review and reinforce concepts.	More than 85% of the parents identified correct answers to 4 of 11 genetic concepts; most knew that “genes are made of DNA,” “genetic risk is the chance of having an inherited (passed down) disease or disorder,” “healthy parents can have a child with an inherited disease,” and “genomic testing of your child’s tumor and healthy tissue may teach you things about (multiple choice responses).”. Baseline understanding of differences between somatic and germline mutations was poor; 31% of parents answered correctly, “nontumor (germline) mutations are in every cell of your body,” and 18% answered correctly, “tumor (somatic) mutations are only found in cancer cells.”	After completion of the 2-visit intervention, correct responses increased significantly for 9 of 11 genetic concepts and overall genetic knowledge; the median percentage of total correct answers improved from 77.8% to 88.9%. The rate of understanding that somatic mutations are only found in cancer cells increased from 18% to 59% and understanding that germline mutations are found in every cell of the body went from 31% to 64%. No association was detected between the change in the overall percentage of correct answers and parental numeracy, literacy, or sociodemographic factors.

^a^GKI: Genetic Knowledge Index.

## Discussion

### Principal Findings

This review reveals important information regarding the current genomic health literacy levels among pediatric populations and the attitudes they hold toward receiving genomic information and decision-making. The findings from this review are valuable in informing the design of digital health platforms, such as the UCAN genomics education platform, that aims to educate young patients with JIA about genomic concepts. We found that age is associated with increased genomic health literacy levels and increased perceived capacity to participate in decision-making regarding genomic information. It was also found that internet-searching is valued by young people and parents for information on diagnoses and symptom management and to fill critical gaps in communication with their HCPs. In addition, we found that adolescents found key differences in receiving genomic information from genetic counselors, compared with HCPs, which helped them contextualize their condition as a part of their identity.

Patient education regarding genomics is an emerging area of research and nearly half of the selected studies describe the goal of assessing genetic or genomic knowledge among the participants. The studies in this review found that genomic health literacy is generally low among patients with genetic conditions and their parents. Several studies in our review found that age plays a significant role in young people’s understanding of genetics [16,37,39] and their decision-making capabilities [12,38]. These findings might reflect the general teaching practices in the United States, Canada, and the United Kingdom, where genetic concepts, such as genetics, inheritance, and DNA, are formally introduced to students during the high school period (15-16 years) [46-48]. A study by Dougherty et al [46] assessing grade 12 students’ understanding of essential genetic concepts across the United States, using core concepts developed by the American Society of Human Genetics as normative benchmarks, found that the states’ understanding of genetic concepts was generally poor, with more than 85% of the states receiving overall scores of *inadequate*. A total of 14% (2/14) of studies in our review evaluated the change in understanding and knowledge of genetic concepts using the GKI among the participants [3,41]. These studies found that the oral presentation of information combined with a visual aid such as a brochure [41] or a children’s book [3] improved understanding among participants. Both studies also indicated that the presence of a nurse to explain genetic information was beneficial in guiding knowledge acquisition among both children and parents. Although both interventions reported an improvement in genetic literacy after the intervention compared with before the intervention, the understanding of some genetic concepts such as the difference between somatic and germline mutations [41] and DNA functionality [3] remained suboptimal after the intervention. Owing to the complex nature of genetic concepts and the general difficulty that children face in understanding the functionality of genes, genetics [3,37], and whole-genome sequencing [39], it may be valuable to limit patient education to high-level information about genomics, genetics, and how they relate to the patient’s disease.

Numerous studies highlighted the importance of internet-searching and seeking emotional support on the web among parents of children with genetic illnesses [33,35,36,39,45]. In general, searching the internet was reported as a key step in knowledge acquisition, improving understanding, and finding treatment options and to fill the gap of social support by finding networks of families with similar experiences. These findings are consistent with research on patients with JIA, who have listed the internet as a source for general information on JIA and emotional support [49,50]. Studies also reported that having a diagnosis played a key role in internet-searching; other research in children undergoing exome sequencing has found that families place significant value on receiving a timely diagnosis, information, and knowledge for rare illnesses [14,51]. The internet has drastically increased parents’ access to the previously privileged health information, potentially changing their expectations and affecting their relationships with HCPs [36,52-54].

eHealth users list the internet as a source of health information but may not always feel comfortable sharing this information with their child’s physician; a study by Tuffrey and Finlay [55] found that 84% of parents who used the internet before a pediatric visit evaluated the information they obtained as useful but only a small proportion of these parents discuss what they found on the internet with their child’s physician. Similarly, several studies in this review reported that parents generally felt dissatisfied with the information they received from their health care practitioners [43], received discouragement from providers to search the internet for information, [33] or felt uncomfortable sharing the information they found on the internet with their physicians [35]. In addition, parents expressed the need for receiving more information from their HCPs and sought additional resources after talking to their HCPs. These findings are similar to the research with patients with JIA; a study by van Dijkhuizen et al [56] found that patients with JIA were most dissatisfied with the low rates of referrals and the information about immunizations, research, and existence of transition of care clinics. It is evident that eHealth resources play a large role in knowledge acquisition for parents of pediatric patients. Future research should aim to find strategies to improve knowledge-sharing among HCPs, patients, and parents; decrease the discouragement of internet-searching from providers; and guide parents toward reliable and credible internet resources for information and emotional support.

Similar to the studies that highlighted the differences in the knowledge of genomic concepts among young children, adolescents, and parents, several studies highlighted the importance of age in decision-making and the preferences related to genetic information [12,38,39]. Although most adolescents wished to be involved in the decision-making process, parents expressed concerns regarding their child’s privacy and capacity to understand genetic information. In general, most participants preferred a shared decision-making model involving the child, parents, and health care practitioners. Several studies exploring decision-making views among patients with chronic illnesses have also reported that a shared decision-making model is preferred by adolescents and their parents [46-48]. In particular, research in the population with JIA has shown that adolescents wish to be a part of the decision-making process and that the parents and providers play a key role in involving children in decision-making and educating them about their disease [57,58].

Research regarding the psychological impact of returning genetic information to children has shown mixed results. A review by Wakefield et al [59] found that serious adverse psychological outcomes such as anxiety, depression, and distress from receiving genetic information were uncommon among children; however, some children experienced interfamilial distress, discrimination, and regret. Research has also shown that receiving genetic information during childhood may allow for early psychological adjustment and the ability to share information with other family members [60]. More research is required to assess the psychological impact and ethical implications of returning genetic information to children, especially for conditions that may not be treatable or modifiable [59]. Therefore, although adolescents prefer to be involved in the decision-making process, a shared decision-making model involving the adolescent, parents, and HCPs may be best suited for decision-making involving the return of genetic information for adolescents with genetic illnesses.

Although only 7% (1/14) of studies explored adolescents’ experiences with genetic counseling [16], it presents important implications for sharing genomic information with young adolescents. Adolescents reported key differences in their experiences talking to a genetic counselor compared with an HCP and felt that learning about genetics, inheritance, and origins of the condition helped them contextualize their condition as a part of their identity and helped them understand their disease. Interestingly, the participants also suggested using videos to describe genomic concepts; a study by Sabatello et al [15] also found that a video format was more effective in increasing self-reported genomic knowledge compared with a pamphlet format. Similar to our findings in the knowledge and decision-making categories, the participants reiterated that age was an important factor in their understanding of genetic information and that high school was a more appropriate period to receive genetic counseling compared with elementary school. However, it is important to consider that 1 study is not representative of the general attitudes that adolescents have toward genetic counseling and more research must be done in the field of genetic counseling and patient education among children and adolescents.

Although the primary purpose of our research was to investigate the knowledge of genomic concepts, in particular, most results from our search returned studies focused on genetic testing, perhaps owing to the novelty of the field of genomic education among pediatric populations. We leveraged a broad search strategy to encompass findings from various fields of genomics health literacy and patient education, which can be leveraged to inform the design of digital health interventions for genomics education. In addition to conducting formative research for the UCAN study, The Centre for Global eHealth Innovation at the University Health Network has developed and conducted usability studies for multiple patient-centric digital applications that aid in the self-management of chronic diseases such as diabetes [61], asthma [62], arthritis [63], prostate cancer [64], and heart failure [65]. The overarching theme among these studies has been to find strategies to engage and educate patients about their chronic illness to facilitate informed decision-making and disease self-management. Thus, the following findings (in no particular order) presented in Textbox 2 could be considered for developing design principles for digital health interventions.

**Key findings for informing the design of patient education digital health interventions.**

**Key findings**

- Providing the patients and parents a high-level overview of genomic concepts relevant to their condition, supplemented by an overview of genetics if applicable to the patient.
- Curating and separating educational content for different age groups (young children, adolescents, and parents) based on their differing capacity to understand genomic information.
- Using visual aids such as illustrations and videos to facilitate engagement.
- Testing the understanding of genomic and genetic concepts without creating the pressure of a test environment, for example, by leveraging quizzes such as the Genetic Knowledge Index before and after providing the patient and parents with educational materials or through a deeper discussion among patients, parents, health care providers or nurses to evaluate the understanding of genomic and genetic concepts.
- Providing a repository of credible and reliable web resources for patients and their parents to seek information relevant to their condition and to seek emotional support in the community.

Most studies in this review were qualitative or mixed methods studies and used methods such as interviews or surveys to gather data from patients, which likely reflects the state of science in pediatric populations and, for most genetic illnesses, remains at the purely exploratory or descriptive level at this time. A limitation of our study is that our understanding of patient education interventions is still limited as only 14% (2/14) of studies leveraged an intervention design to evaluate the change in genetic knowledge of the participants [3,41]. A key limitation of the intervention by Newcomb et al [3] was that the participants were not asked to complete the GKI questionnaire after the test and the understanding of concepts such as *DNA* and *genes* was assessed qualitatively among the participants, which may have led to subjectivity and bias in reporting of posttest results. Another limitation was that the study by Johnson et al [41] investigated learning in parents with a mean age of 37.5 years. Thus, the findings from the study by Johnson et al [41] may not be applicable to inform the design of patient education interventions for children. More research that incorporates intervention methodology to evaluate participants’ genomic health literacy levels and attitudes is required to evaluate the best tools for improving genomic literacy in children.

Another limitation of our review is that 29% (4/14) of the included studies investigated the understanding of genetic concepts among populations that do not have any specified disease [12,37-39]. Although it is valuable to gauge the level of genetic knowledge and understanding in the general public, the knowledge and attitudes toward receiving and seeking genomic information may be very different for families in which a family member has a medical condition compared with populations that are not diagnosed with a genetic condition. Moreover, of the 14 studies, 8 (57%) included parent study populations and only 6 (43%) included child and adolescent populations, which may be because of the ethical barriers that currently exist in returning genetic information to children or evaluating their role in genetic decision-making [20,21].

In addition, a key consideration is that we used a broad definition for the terms *genetic* and *genomic* information for the inclusion criteria, which align with the definition of genomic health literacy: “basic knowledge of genetic and genomic concepts and the capacity to obtain, process, understand, and use genomic information for health-related decision-making” [6]. The rationale behind using a broad definition and including study populations with a broad range of genetic and genomic illnesses was to capture a diverse range of ongoing initiatives that aim to improve genomic health literacy. However, the key findings to curate design principles for digital health interventions are considerations to create digital health interventions for all pediatric populations with genetic conditions and not specific to a singular genetic illness.

Furthermore, the inclusion criteria were limited to studies from the past 12 years to capture lead practices and emerging trends in digital health interventions and genomics. However, a larger sample of relevant studies on patient education and genomic health literacy may have been captured if studies older than 12 years were also included.

Moreover, the ability to cross-culturally compare the knowledge and understanding of genetic concepts was limited because most of the studies were conducted in the United States and only studies published in English were included in this review. This may have also led to bias when reporting results of genomic knowledge among children and their parents as educational systems vary across different countries.

### Conclusions

Our review indicates that although pediatric patients and their parents have a positive attitude toward learning genomic information, we still have little knowledge about the genomic health literacy levels among children and adolescents, their capacity to understand genomic concepts, how this information can be presented, and what best practices can be leveraged to design digital health patient education interventions for genomic education. The rise of the personalized and precision medicine approach demands more patient and parent engagement, and it is the medical world’s mandate to develop tools that improve patient education on disease knowledge and genomic factors involved. Thus, there is a need for studies that examine the genomic health literacy and modalities to inform the design of digital interventions that aim to educate adolescents and children with pediatric illnesses about genomics.

## Appendix

Multimedia Appendix 1 Search strategy.

Multimedia Appendix 2 Data extraction table.

Multimedia Appendix 3 PRISMA (Preferred Reporting Items for Systematic Reviews and Meta-Analyses) diagram.

## Abbreviations

GKI: Genetic Knowledge Index
HCP: health care provider
JIA: juvenile idiopathic arthritis
PRISMA-ScR: Preferred Reporting Items for Systematic Reviews and Meta-Analyses extension for Scoping Reviews
UCAN: Understanding Childhood Arthritis Network
