# Supplementary material for: Genomic Health Literacy Interventions in Pediatrics: Scoping Review
Source: J Med Internet Res. 2021 Dec 24;23(12):e26684. doi: 10.2196/26684 (PMC8742210; doi:10.2196/26684)
Supplement: Multimedia Appendix 1 [file jmir_v23i12e26684_app1.pdf]

Figure 1. Search Strategy

MEDLINE	EMBASE	SCOPUS	CINAHL
Concept 1: Pediatrics			
1. pediatric.mp. or exp Pediatrics/<br>2. paediatric*.tw,kf.<br><br>3. JIA.tw,kf.<br>4. ((pediatric or paediatric) adj5 illness).tw,kf.<br>5. "juvenile idiopathic arthritis".tw,kf.<br>6. ((young or adolescen* or child*) adj5 illness).tw,kf.<br>7. autoimmune disease.mp. or exp Autoimmune Diseases/	1. pediatric.mp. or exp pediatrics/<br>2. paediatric*.tw,kw.<br><br>3. JIA.tw,kw.<br>4. ((pediatric or paediatric) adj5 illness).tw,kw.<br>5. "juvenile idiopathic arthritis".tw,kw.<br>6. ((young or adolescen* or child*) adj5 illness).tw,kw.<br><br>7. autoimmune disease.mp. or exp autoimmune disease/	TITLE-ABS-KEY ((pediatric*) OR (paediatric*) (JIA) OR ((pediatric or paediatric) w/5 illness) OR ("juvenile idiopathic arthritis") OR ((young or adolescen* or child* w/5 illness) OR ("autoimmune disease"))	(MH "Pediatrics+")<br><br>(MH "Autoimmune Diseases+")<br><br>(TI (paediatric*) OR ((paediatric or pediatric) n5 illness) OR ("juvenile idiopathic arthritis") OR ((young or adolescen* or child* n5 illness)) OR (AB ((pediatric n5 illness) OR ("juvenile idiopathic arthritis") OR ((young or adolescen* or child* n5 illness))
Concept 2: Patient education			
7. Patient education.mp. or exp Patient education as Topic/<br>8. ((patient or young or adolescen* or child*) adj5 (educat* or learn* or knowledge or literacy or info*)).tw,kf.<br>9. "health knowledge".tw,kf.	7. Patient education.mp. or exp patient education/<br><br>8. ((patient or young or adolescen* or child*) adj5 (educat* or learn* or knowledge or literacy or info*)).tw,kw.<br>9. "health knowledge".tw,kw.	TITLE-ABS-KEY (("patient education") OR ((patient or young or adolescen* or child*) w/5 (educat* or learn* or knowledge or literacy or info*)) or "health knowledge")	(MH "Patient Education+")<br><br><br>(TI (("patient education") OR ((patient or young or adolescen* or child*) n5 (educat* or learn* or knowledge or literacy or info*)) or "health knowledge")) OR (AB (("patient education") OR ((patient or young or adolescen* or child*) n5 (educat* or learn* or knowledge or literacy or info*)) or "health knowledge"))
Concept 3: Genomics			
10. exp Genomics/	10. exp Genomics/	TITLE-ABS-KEY((genetic* or	(MH "Genomics+")

11. (genetic* or genomic* or genom* or biologic*).tw,kf.<br>12. exp Genetic Counseling/<br>13. (genetic adj5 (counselling or counseling)).tw,kf.<br><br>14. "genetic testing".tw,kf.<br>15. "genom* sequencing".tw,kf.	11. (genetic* or genomic* or genom* or biologic*).tw,kw.<br><br>12. genetic counselling.mp. or exp genetic counseling/<br><br>13. (genetic adj5 (counselling or counseling)).tw,kw.<br><br>14. "genetic testing".tw,kw.<br>15. "genom* sequencing".tw,kw	genomic* or genom* or biologic*) or ("genetic counselling") or ("genetic w/5 counselling or counseling") or ("genetic testing") or ("genom* sequencing"))	(MH "Genetic Counseling")<br><br>(TI ((genetic* or genomic* or genom* or biologic*) or ("genetic counselling") or ("genetic n5 counselling or counseling") or ("genetic testing") or ("genom* sequencing"))) OR (AB (((genetic* or genomic* or genom* or biologic*) or ("genetic counselling") or ("genetic n5 counselling or counseling") or ("genetic testing") or ("genom* sequencing")))
Combos + Limits			
16. 1 or 2 or 3 or 4 or 5 or<br>17. 7 or 8 or 9<br>18. 10 or 11 or 12 or 13 or 14 or 15<br>19. 16 and 17 and 18<br><br>20. limit 19 to english<br><br>21. limit 20 to last 12 years	16. 1 or 2 or 3 or 4 or 5 or 6<br>17. 7 or 8 or 9<br>18. 11 or 12 or 13 or 14 or 15<br>19. 16 and 17 and 18<br><br>20. limit 19 to english<br><br>21. limit 20 to last 12 years	English/2008-2020	English/2008-2020
