# Supplementary material for: Genomic Health Literacy Interventions in Pediatrics: Scoping Review
Source: J Med Internet Res. 2021 Dec 24;23(12):e26684. doi: 10.2196/26684 (PMC8742210; doi:10.2196/26684)
Supplement: Multimedia Appendix 3 [file jmir_v23i12e26684_app3.pdf]

Figure 2. Prisma diagram

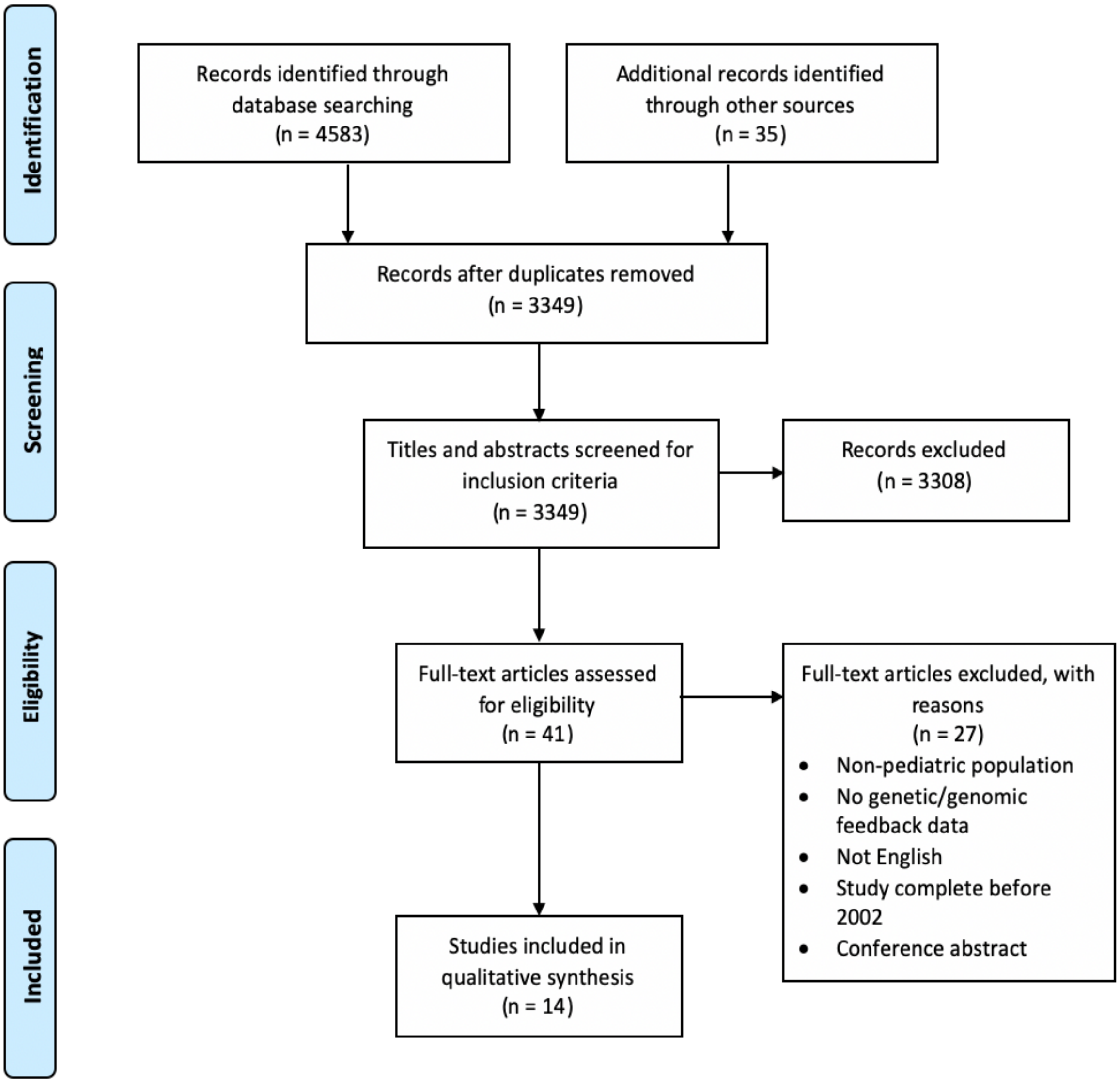

From: Moher D, Liberati A, Tetzlaff J, Altman DG, The PRISMA Group (2009). Preferred Reporting Items for Systematic Reviews and Meta-Analyses: The PRISMA Statement. PLoS Med 6(7): e1000097. doi:10.1371/journal.pmed1000097
